# Assessing EHR use during hospital morning rounds: A multi-faceted study

**DOI:** 10.1371/journal.pone.0212816

**Published:** 2019-02-25

**Authors:** Shiri Assis-Hassid, Barbara J. Grosz, Eyal Zimlichman, Ronen Rozenblum, David W. Bates

**Affiliations:** 1 Harvard John A. Paulson School of Engineering and Applied Sciences, Cambridge, MA, United States of America; 2 Brigham and Women’s Hospital, Boston, MA, United States of America; 3 Harvard Medical School, Boston, MA, United States of America; 4 Sheba Medical Center, Ramat-Gan, Israel; 5 Sackler Faculty of Medicine, Tel Aviv University, Tel Aviv, Israel; 6 Harvard T.H. Chan School of Public Health, Boston, MA, United States of America; Brown University, UNITED STATES

## Abstract

**Background:**

The majority of U.S hospitals have implemented electronic health records (EHRs). While the benefits of EHRs have been widely touted, little is known about their effects on inpatient care, including how well they meet workflow needs and support care.

**Objective:**

Assess the extent to which EHRs support care team workflow during hospital morning rounds.

**Design:**

We applied a mixed-method approach including observations of care teams during morning rounds, semi-structured interviews and an electronic survey of hospital inpatient clinicians. Structured field notes taken during observations were used to identify workflow patterns for analysis. We applied a grounded theory approach to extract emerging themes from interview transcripts and used SPSS Statistics 24 to analyze survey responses.

**Setting:**

Medical units at a major teaching hospital in New England.

**Results:**

Data triangulation across the three analyses yielded four main findings: (1) a high degree of variance in the ways care teams use EHRs during morning rounds. (2) Pervasive use of workarounds at critical points of care (3) EHRs are not used for information sharing and frequently impede intra-care team communication. (4) System design and hospital room settings do not adequately support care team workflow.

**Conclusions:**

Gaps between EHR design and the functionality needed in the complex inpatient environment result in lack of standardized workflows, extensive use of workarounds and team communication issues. These issues pose a threat to patient safety and quality of care. Possible solutions need to include improvements in EHR design, care team training and changes to the hospital room setting.

## Introduction

EHRs are intended to improve various aspects of care, including patient safety, clinical decision-making and information exchange [1–3]. They may also represent a cost-effective tool for improving quality of care [4]. Many challenges remain to effective EHR use, however, including prolonged documentation time, interference with communication, usability difficulties, lack of cognitive support, failure to support the clinical workflow and low user satisfaction [5–9]. Nonetheless, financial incentives of meaningful use criteria and the Medicare incentive program [10] have led to high EHR adoption rates in the U.S., with 96% of hospitals having certified EHR technology [42]. Although these rates represent a major milestone, there is little evidence that EHRs are improving the quality of inpatient care [11,12]. Even though efficiency seems to have improved [13], usability appears to be mixed at best [14] and EHRs' ability to provide proper support for care team workflow has not yet been established. Anecdotal reports suggest that EHRs may be contributing to clinician burnout [15,16].

This study provides an assessment of EHR use in an inpatient setting. We apply a mixed-method approach to investigate the ways EHRs are used during morning rounds. We assess the extent to which they support care team workflow and delivery of patient care and identify existing problems.

## Methods

### Setting

This study was conducted at a major teaching hospital in New England with over 700 beds and it was approved by its IRB. Clinicians and patients who participated in this study provided their informed consent. The hospital implemented EPIC’s EHR (Verona, Wisconsin) across all units in May 2015. To cover a wide range of medical conditions, we focused on care teams and patients in the medical units. Care teams typically comprised an attending clinician, resident, 1–2 interns, medical student and pharmacist.

### Data collection and analysis

To examine EHR use in inpatient care we used three complementary methods: (1) observations during morning rounds; (2) semi-structured interviews; and (3) an electronic survey. Participants were selected based only on their involvement in morning rounds and their availability. After rounds were completed, clinicians were approached and asked to participate in an interview. Results from observations and interviews informed the design of a user preference survey. This survey was distributed to inpatient clinicians, to validate with a larger sample findings from observations and interviews.

#### Recruitment process and participants

We approached attendings that were scheduled for morning rounds by email. We provided a study fact sheet (S1 Appendix) that included a detailed description of the study’s objective, the researcher conducting the observations, and details of the investigators involved in the study. The study fact sheet also described the interview portion of the study and participants were informed that upon their consent they might be asked to participate in an interview. The email requested the attending’s consent to take part in their morning round and observe them and their care team. Following this initial stage of consent provided by attendings, upon arrival to the medical unit, we approached the care team of each of the observed rounds to receive their consent to be observed. It was agreed by the research team that the researcher would ask care teams’ for their consent directly and not the attending to avoid concerns regarding hierarchical relationships. The study fact sheet was provided to care teams with a verbal explanation. To avoid care team burden, we utilized verbal consent. All attendings and care teams that were approached in this study agreed to be observed.

The survey was created by the authors and sent to three attending clinicians as a pilot, to ensure that the wording is correct and that the questions are clear. To recruit participants for the survey, we obtained list of clinicians that work at the hospital’s medical units. The survey was sent by email to the clinicians. Reminders were sent to all participants three times until no additional responses were recorded and we concluded that additional responses could not be collected. We administered the survey using Redcap, which is an accepted and well-known survey tool at the hospital.

#### Observations

Observations were carried out by the first author of this paper (SAH) with peer-debriefing following each observation session. We observed care teams during their morning rounds. The observing researcher took structured notes to record EHR use before entering the patient’s room, in the patient’s room and after leaving the room. Items for observation included: which care team clinicians used the EHR, the device used to access the EHR, EHR functional use in the room, and use of printouts in the patient’s room. Analysis of the field notes yielded workflow diagrams that allowed identifying different patterns of EHR use.

#### Semi-structured interviews

We interviewed inpatient clinicians in the medical units. Our semi-structured interview guide (S2 Appendix) was designed to capture care teams’ perspectives on integrating EHR use in their workflow and possible challenges they faced. Questions were designed to yield an in-depth understanding of inpatient clinical workflow, information needs during rounds and EHR use patterns. Interviews were recorded and transcribed.

Interview data were analyzed using the constant comparative approach [17,18]. Consistent with qualitative research methodology [19], the interview guide was established without a priori themes or codes for data classification. We used Dedoose software (http://www.dedoose.com/) to review each transcript line by line documenting comments and quotations with similar meaning into a list of distinct codes. During the coding process, new interview data were compared with previous quotes under the same code name. Transcripts were reviewed several times to ensure that all relevant data were properly assembled under a defined code and until a full coding scheme was established. The coding scheme was shared with two of the leading investigators (EZ, BJG) through a series of group meetings to discuss the quotes, their assigned codes, and the coding scheme until consensus was reached. The coding scheme was applied to the full set of transcripts (S3 Appendix). Coded excerpts were then grouped to identify emerging themes and their relationships. The grouping process was also conducted iteratively; we carried out discussions on the meaning of the codes and excerpts and possible themes until consensus was reached on which code/excerpt should be assigned to each theme.

#### Survey

To augment the observations and interviews and substantiate the results on a larger sample, we surveyed 306 clinicians at the hospital’s medical units. Participants were asked to rate 18 statements about EHR use in their workflow on a discrete scale: never, sometimes, most of the time, always (S4 Appendix). We conducted a between-group analysis to identify differences in behavior that might be attributed to clinical role (attending clinician, resident, intern, PA).

### Ethics statement

This study was approved by the Partners Human Research Committee. The investigator conducting observations and interviews met with the teams before rounds began (without the attending's presence), provided the care team with the fact sheet, explained the research purpose as well as the observation and interview portions of the study and asked for their verbal consent. The care teams were assured that they can refuse to be observed/interviewed. The verbal consent process was documented in the observation notes and on the audio recording of the interviews in accordance with the Partners Human Research Committee’s policy regarding verbal informed consent. The verbal consent process was approved by the committee and was intended to avoid burdening participants with documenting their consent in writing during their busy routine.

## Results

Participant characteristics are summarized in Table 1. We observed 12 care teams for a total of 50 hours and interviewed 13 clinicians including: 4 attending clinicians, 3 residents, 1 intern and 5 Physician Assistants (PAs). Our survey yielded a 30% response rate of 91 respondents: 32 attending clinicians, 26 residents, 20 interns and 13 PAs. We analyzed results of each method, and then compared the three analyses to identify recurring themes. This analysis yielded the following themes: (1) variance in EHR use patterns during morning rounds; (2) use of workarounds in care team workflow; (3) use of EHRs for information sharing and its impact on intra-care team communication; and (4) challenges presented by EHR design and usability. In the following sections we describe each theme and provide supporting findings from the observations, interviews and survey.

**Table 1:** Participant characteristics.

**Observations**		
*n (participants)*	*n (hours of observations)*	*Gender*
12 care teams	50	Female attendings = 17%
		Male attendings = 83%
		Teams were always comprised of female and male clinicians
**Interviews**		
*n (participants)*	*n (clinician type)*	*Gender*
13	Attending clinicians– 4	Female = 46%
	Residents– 3	
	Interns– 1	Male = 54%
	PAs– 5	
**Survey**		
*n (participants)*	*n (clinician type)*	*Age Range*
91	Attending clinicians– 32	25–34–56
	Residents– 26	35–44–26%
	Interns– 20	45–54–15%
	PAs– 13	55–64–2%

### Theme 1: Variance in EHR use patterns during morning rounds

**Fig 1:**
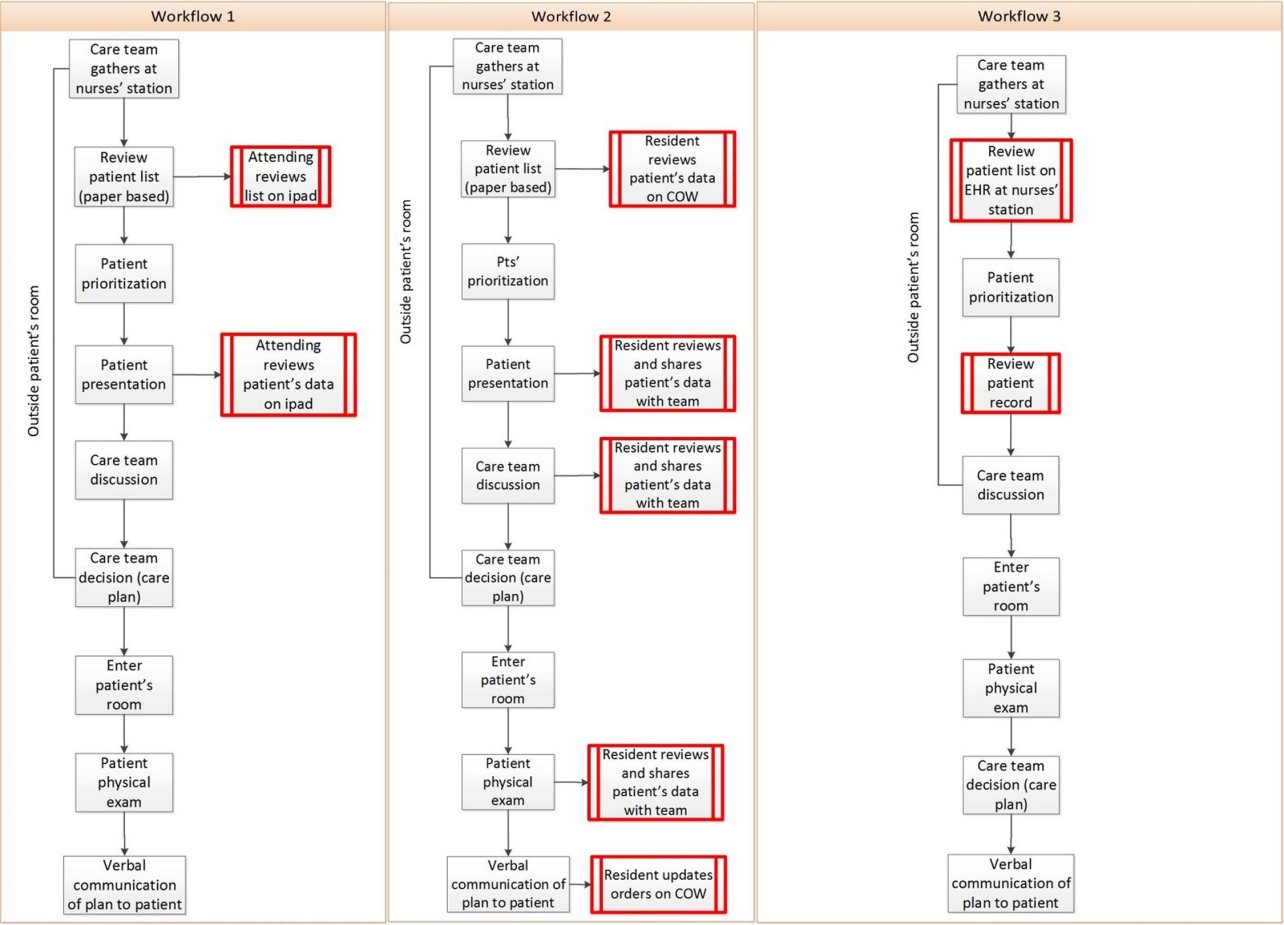
Example workflows showing variation in EHR use patterns during morning rounds. Colored squares identify the stages at which the EHR was used. Different colors represent different EHR users in the workflow.

We found a high degree of variance among care teams’ patterns of EHR use in their workflow. Variation appeared in the type of clinician using the EHR, the stage at which the EHR was used and the device used. To illustrate this variation, Fig 1 shows an example of three workflows we observed. Red boxes indicate the stage at which the EHR was used. In workflow 1 an iPad was used by the attending clinician to review patient data outside the patient’s room. In workflow 2 a computer on wheels (COW) was used by the resident, and it was used both inside and outside the patient’s room. In workflow 3 the desktop at the nurses’ station was used by the attending clinician and intern outside the patient’s room.

Table 2 shows the survey results for the different clinician types’ EHR use patterns during morning rounds. These results align with our observations in that they indicate variance in the clinician type using the EHR, the stage at which the EHR was used and the device used. Most clinicians reported that they always use the EHR before entering the patient’s room, but only sometimes in the room. With respect to EHR use before rounds, most clinicians reported they always use the EHR before entering the patient’s room, but only sometimes when in the patient’s room. When asked about EHR use after leaving the patient’s room, clinician types varied in their responses, 40.6% of the attending clinicians and 50% of the residents reported using the EHR after leaving the patient’s room sometimes, whereas 50% of the interns and 53.8% of the PAs reported always using the EHR after leaving the room. With respect to the device used during rounds, most clinicians reported not using iPads during rounds at all, though iPads are equally available to clinicians during rounds. Most clinicians also reported using a Smartphone or the bedside computer only sometimes, though most clinicians have smartphones and use them regularly. When asked about using a COW during rounds, responses varied among clinician type. 68.8% of the attending clinicians and 55% of the interns reported that they use a COW sometimes, 50% of the residents reported using a COW most of the time, and 53.8% of the PAs reported they never do. COW availability may vary in the medical units and it is important to note that they require charging in advance and cannot be transported between different floors. Most clinicians reported that they use the desktop at the nurses’ station most of the time.

**Table 2:** EHR use patterns during morning rounds: Survey responses based on clinician type.

Survey question	Clinician type	Response distribution			
		Never	Sometimes	Most of the time	Always
I usually use the EHR before entering the patient’s room	Attending (n = 32)	0.0%	28.1%	31.3%	40.6%
	Resident (n = 26)	0.0%	3.8%	26.9%	69.2%
	Intern (n = 20)	0.0%	5.0%	20.0%	75.0%
	PA (n = 13)	0.0%	0.0%	15.4%	84.6%
I usually use the EHR while in the patient’s room	Attending (n = 32)	3.1%	81.3%	15.6%	0.0%
	Resident (n = 26)	3.8%	76.9%	19.2%	0.0%
	Intern (n = 20)	10.0%	75.0%	15.0%	0.0%
	PA (n = 13)	7.7%	84.6%	7.7%	0.0%
I usually use the EHR after leaving the patient’s room	Attending (n = 32)	0.0%	40.6%	28.1%	31.3%
	Resident (n = 26)	3.8%	50.0%	11.5%	34.6%
	Intern (n = 20)	0.0%	35.0%	15.0%	50.0%
	PA (n = 13)	0.0%	23.1%	23.1%	53.8%
When I review the patient’s medical record on the EHR I use an iPad	Attending (n = 32)	84.4%	6.3%	9.4%	0.0%
	Resident (n = 26)	88.5%	11.5%	0.0%	0.0%
	Intern (n = 20)	100.0%	0.0%	0.0%	0.0%
	PA (n = 13)	92.3%	7.7%	0.0%	0.0%
When I review the patient’s medical record on the EHR I use a Smartphone	Attending (n = 32)	18.8%	68.8%	12.5%	0.0%
	Resident (n = 26)	23.1%	65.4%	7.7%	3.8%
	Intern (n = 20)	20.0%	65.0%	10.0%	5.0%
	PA (n = 13)	15.4%	76.9%	7.7%	0.0%
When I review the patient’s medical record on the EHR I use a COW	Attending (n = 32)	21.9%	68.8%	6.3%	3.1%
	Resident (n = 26)	0.0%	42.3%	50.0%	7.7%
	Intern (n = 20)	0.0%	55.0%	35.0%	10.0%
	PA (n = 13)	53.8%	46.2%	0.0%	0.0%
When I review the patient’s medical record on the EHR I use the desktop at the nurses’ station	Attending (n = 32)	0.0%	40.6%	43.8%	15.6%
	Resident (n = 26)	0.0%	38.5%	50.0%	11.5%
	Intern (n = 20)	0.0%	35.0%	60.0%	5.0%
	PA (n = 13)	0.0%	30.8%	61.5%	7.7%
When I review the patient’s medical record on the EHR I use the desktop at the bedside computer	Attending (n = 32)	9.4%	81.3%	9.4%	0.0%
	Resident (n = 26)	15.4%	73.1%	11.5%	0.0%
	Intern (n = 20)	15.0%	80.0%	5.0%	0.0%
	PA (n = 13)	7.7%	76.9%	15.4%	0.0%

### Theme 2: Use of workarounds in care team workflow

Workarounds have been defined by Koppel et al. as ‘actions that do not follow explicit or implicit rules, assumptions, workflow regulations, or intentions of system designers. They are nonstandard procedures typically used because of deficiencies in system or workflow design’[20]. Flanagan et al. (2013) [7] extend this definition to include real or perceived limitations in a technical system [7,21]. Workarounds may include circumvention of health IT processes or procedures as a result of user interface flaws or human–technology integration factors, or necessary actions to complete a task [22]. We suggest that the integration of EHRs into the clinical workflow is intended to improve data accuracy during the process of morning rounds, whereas our observations and interviews reveal that the EHR needed to be supplemented by processes that involve information being transmitted outside of the system and in addition to using it.

Workarounds that were documented during observations and interviews included extended use of handwriting, emails and verbal discussions. Printouts of patient summary reports were used to add information from the EHR in handwriting, such as: vital signs and recent lab results. One resident explained that using handwriting helps process patient information: “*The information that I have in handwriting*, *I write down all the morning labs based on what I see on the EHR*. *I do this by hand because it's easier for me to process the information when I'm writing that for myself*. *Then I also have mostly check boxes that are to-dos*. *These are tasks that need to be done during the day*. *Whether it's talking to a consultant*, *making sure that this lab or this order is made in the EHR*, *this medication is started*, *or follow up on this result” (Resident #2)*. In addition to the use of handwriting as a cognitive aid for clinicians, it is also perceived as more useful than using the EHR during rounds, this point was discussed by one of the PAs: “*I like to write my lab results on this page*. *I find that looking at them on the computer*, *I don't tend to recall them as well*, *I don't seem to recognize them*, *or act upon them unless it's in front of me*.*” (PA #1)*.

**Fig 2:**
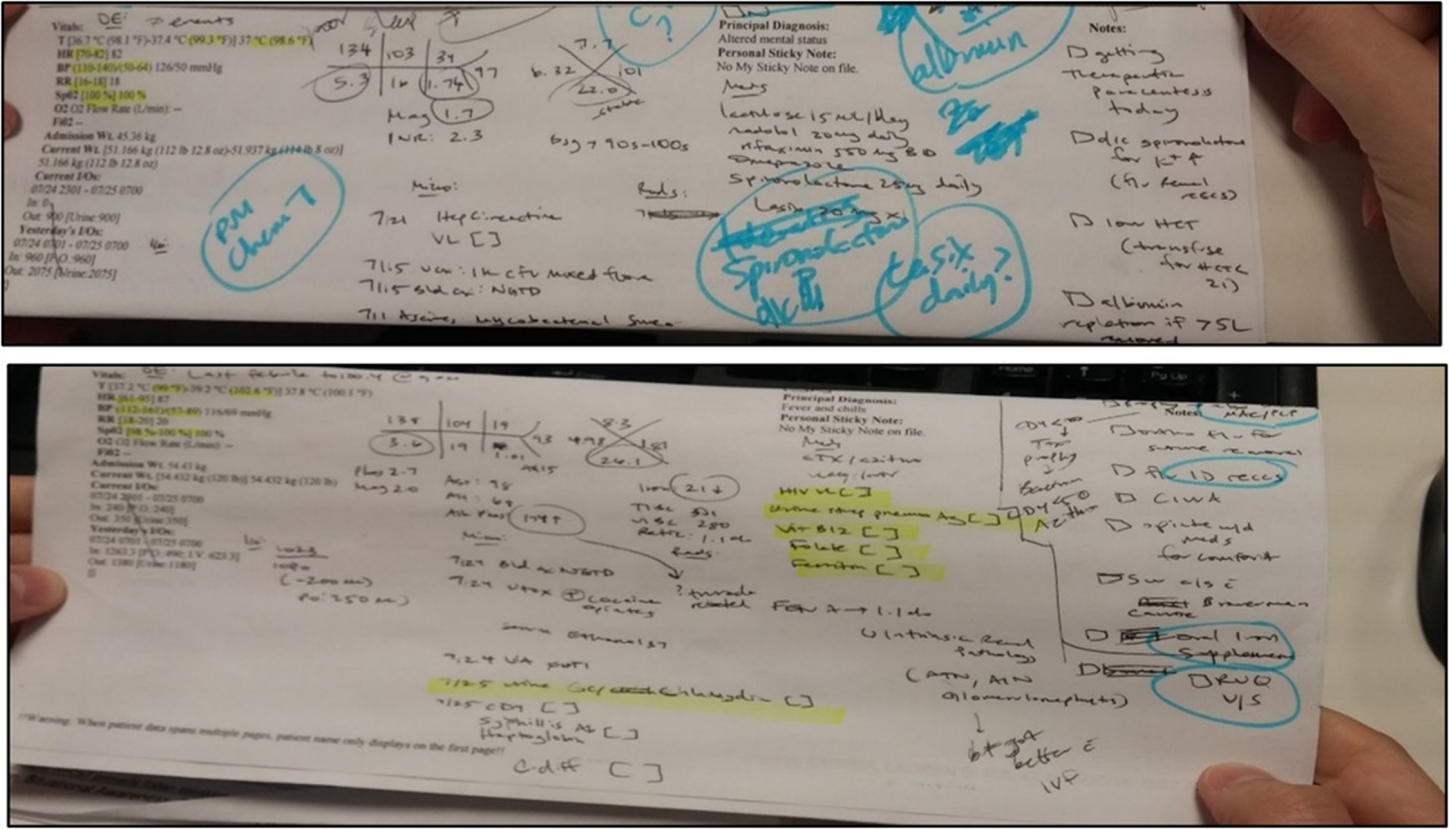
Use of handwriting on patient summary report during rounds.

Patient summary reports were also used during rounds to track the patients being visited, document tasks and reminders, and update lab results that would later be typed into the patient’s progress notes, as one attending described: “*I print out for myself just a basic list that has all the kind of just the patients names*, *it has their PCP on it*, *and kind of which intern or PA has the patient*, *and sometimes jot down some notes onto that*, *if there are*, *as I’m doing my physical exam*, *if there are particular things that I’m going to note*, *want to document*, *but not going to remember*, *who I'd remember*, *who didn't*, *I’ll document that and if there are kind of key parts of the plan that I’m like*, *“Wait*, *I’m going to forget*,*” I’ll put that on there as well”(Attending #4)*. Fig 2 shows an example of use of handwriting on a patient summary report during rounds.

We also observed various workarounds during the handoff process, both before and after morning rounds. Email and verbal discussions were used to convey important information and overnight events regarding patients. For example, updates on events that took place during rounds monitoring and debriefing after rounds were carried out either verbally through email. This type of workaround was also discussed during the interview, for example one resident explained: “*After we finish rounds*, *what I actually try to do is verbally go over all the checkboxes for the day with my sub-interns or if they were my interns*, *I'd do the same thing*.*” (Resident #1)*.

### Theme 3: EHR use for information sharing and its effect on intra-care team communication

We observed care team sharing of EHR information both verbally and visually during morning rounds. Overall, such sharing of EHR information was observed sometimes before entering the patient’s room, but rarely in the room. Inside the room, we found that when the attending clinician was using the EHR, s/he usually chose to use the bedside computer. The positioning of the computer in the room prevented the clinician from facing the rest of the team. Furthermore, during computer use, the clinician was focused more on the screen than on interacting with the care team.

This difficulty of balancing computer use and care team communication was raised during interviews with care team clinicians. As described by one attending clinician: *“If the information was presented more user friendly way*, *then maybe we can have it up*. *I don't have to be as focused on trying to find a piece of information that I need to progress the patient's care*. *It's a little bit of a balance back and forth*, *trying to figure out*, *how much I should be looking at that (the EHR) versus how much I should be tuned into listening*.*” (Attending #2)*. During interviews, several clinicians raised concerns about EHR use impeding care team communication because it distracts the clinician in the patient’s room: *“When I’m working with the attending and they’re not looking at me*, *and they’re looking at the screen while I’m talking*. *It feels like they’re not listening*, *and they’re probably not*. *I’m definitely very aware of it and I try to make the best of something that is difficult and probably new for the patient*.*” (PA #5)*.

Table 3 shows the survey results for the different clinician types’ EHR use for information sharing and its effect on intra-care team communication. According to the survey results, most attending clinicians only sometimes verbally share EHR information with the care team, whereas most residents, interns and PAs always do so.

When it comes to visually sharing EHR information, most clinicians responded that they do so sometimes. When asked if EHR use interrupts care team communication, responses varied among clinician types. Most attending clinicians and interns responded that EHR use never interrupts care team communication, while most residents and PAs responded that it does sometimes.

**Table 3:** EHR use for information sharing and its effect on intra care team communication: Survey responses based on clinician type.

Survey question	Clinician type	Response distribution			
		Never	Sometimes	Most of the time	Always
When I use the EHR during rounds I verbally share data from the patient’s record with the care team	Attending (n = 32)	0.0%	43.8%	31.3%	25.0%
	Resident (n = 26)	0.0%	7.7%	34.6%	57.7%
	Intern (n = 20)	0.0%	10.0%	40.0%	50.0%
	PA (n = 13)	0.0%	15.4%	23.1%	61.5%
When I use the EHR during rounds I visually share data from the patient’s record with the care team	Attending (n = 32)	6.3%	75.0%	18.8%	0.0%
	Resident (n = 26)	0.0%	57.7%	38.5%	3.8%
	Intern (n = 20)	10.0%	55.0%	25.0%	10.0%
	PA (n = 13)	7.7%	61.5%	30.8%	0.0%
When a care team member uses the EHR during rounds I find that it interrupts communication within the care team	Attending (n = 32)	53.1%	40.6%	6.3%	0.0%
	Resident (n = 26)	38.5%	57.7%	3.8%	0.0%
	Intern (n = 20)	60.0%	40.0%	0.0%	0.0%
	PA (n = 13)	38.5%	61.5%	0.0%	0.0%

### Theme 4: Challenges in EHR design and usability

Design and usability concerns with EHR were mentioned repeatedly in interviews. Clinicians raised concerns about access and presentation of information. For example one attending clinician mentioned: “*It's not really presented in a way that's useful*. *I always have to click twenty different places to access something*. *To look at results I can do it in twenty different ways*. *Some prefer doing it one way*, *some prefer doing it another way*. *That's a little frustrating*.*” (Attending #2)*. Others discussed processes that are difficult to perform with the EHR, including the handoff process: “*My last service*, *I had two residents and we had to pass off to each other*. *We found the hand-off thing so annoying*, *that we actually resorted to calling each other every night and giving verbal updates of what happened during the day and then we would just go on the checkboxes*.*” (Resident #1)*. Another concern was that the EHR does not provide integration of the patient’s data necessary for the care delivery: “*So*, *in order to get a picture of something*, *if I need one piece of data that's a lab value and one thing that’s a flow sheet and one thing that’s a radiology thing and one thing that’s an order and one thing that the nurse enters and one thing that the physical therapist enters and one thing that the physician enters*, *hard*. *Very very hard*, *it doesn’t integrate well*.*” (Attending #1)* and “*If the information was presented more user friendly way*, *then maybe we can have it up*. *I don't have to be as focused on trying to find a piece of information that I need to progress the patient's care” (Attending #2)*.

Our survey addressed additional workflow aspects of EHR use, including EHR usefulness for synchronizing the care team, for efficiency during rounds and for teaching purposes. Most clinicians responded they find EHR useful for synchronizing the care team regarding patients and for teaching purposes sometimes. Attending clinicians’ responses on the EHR’s role in efficiency during rounds were inconsistent, with nearly half of them reporting that it is useful only sometimes, and the other half reporting that it is useful most of the time. Residents reported that they find the EHR useful for efficient rounding only sometimes, whereas most interns and PAs find it useful most of the time. Table 4 shows the survey results for EHR design and usability challenges according to the different clinician types.

**Table 4:** Challenges in EHR design and usability: Survey responses based on clinician type.

Survey question	Clinician type	Response distribution			
		Never	Sometimes	Most of the time	Always
I find that using the EHR during rounds is useful for synchronizing the care team regarding patients	Attending (n = 32)	3.1%	31.3%	46.9%	18.8%
	Resident (n = 26)	0.0%	30.8%	57.7%	11.5%
	Intern (n = 20)	5.0%	20.0%	55.0%	20.0%
	PA (n = 13)	0.0%	23.1%	61.5%	15.4%
I find that using the EHR during rounds is useful for being efficient during rounds	Attending (n = 32)	3.1%	43.8%	43.8%	9.4%
	Resident (n = 26)	0.0%	38.5%	30.8%	30.8%
	Intern (n = 20)	10.0%	5.0%	55.0%	30.0%
	PA (n = 13)	7.7%	15.4%	69.2%	7.7%
I find that using the EHR during rounds is useful for teaching purposes	Attending (n = 32)	15.6%	62.5%	18.8%	3.1%
	Resident (n = 26)	11.5%	57.7%	26.9%	3.8%
	Intern (n = 20)	5.0%	45.0%	45.0%	5.0%
	PA (n = 13)	0.0%	53.8%	38.5%	7.7%

## Discussion

The objective of this study was to provide a comprehensive view of the different ways EHRs are integrated into care team workflows. We found a high variance in EHR use patterns during morning rounds and extensive use of workarounds. We also found that information from the EHR is seldom shared among care team clinicians while in the patient’s room. These finding may be at least partly explained by existing challenges in EHR design and usability. Negative effects of EHR-based technologies on clinical practice, organizational culture, medical education and patient-clinician communication have been well documented in the literature [23–26]. They include new types of errors, modification of the clinical workflow and changes in clinicians’ cognitive behaviors [2,27–32].

Our findings indicate that the EHR is not regularly used in patients’ rooms as part of the workflow. When it is used in the room, verbal and visual sharing of EHR information among care team members are rare. Visual sharing of the screen, whether it is an iPad, COW or bedside computer, appeared technically difficult in the current hospital room setting. Screen location, screen size, and the available technology do not facilitate a shared view of the EHR. Recognizing the importance of effective communication and teamwork for delivery of high quality and safe patient care [33,34], several medical team training programs have set out to enhance communication between team members [35–37]. These programs, however, do not address the ways EHR use affects care team dynamics and communication or its potential as an information-sharing tool. Our observations and interviews suggest that such an influence clearly exists and, therefore, current programs must account for the role of the EHR in the teams’ work and communication in order to improve patient care and patient safety.

Observations and interviews revealed extensive use of workarounds. Some workarounds, such as handwritten notes were used as a cognitive aid for clinicians. Others were used due to lack of system support. Studies have shown that the format and layout of paper records are critical to the clinicians’ ability to search, read and assess relevant information. The ability to mark-up important findings is important to the cognitive processing of clinical information and could be lost when working directly in the EHR [38]. Moreover, it has been suggested that electronic note taking is less effective than longhand note taking and that it impairs learning due to shallower information processing [39]. However, the extensive use of handwriting as well as other observed workarounds pose a threat to quality of care and patient safety, as they could potentially cause overlooking important information and result in lack of synchronization between care team members. Some workarounds might be addressed through better system design and Human-Computer Interaction (HCI). For example, it has been found that clinicians using comprehensive EHRs are vulnerable to information overload, which might lead them to miss important information [40]. The HCI literature addresses the influence of information overload, which is associated with impeding cognition and thus impairing objective decision making [41]. One solution suggested in the literature is visualization of data which could be used to mitigate the effect of information overload. Presenting data in the form of graphs has been found to reduce such overload and improve decision making outcome as opposed to the use of tables [42]. Other solutions may involve the incorporation of handwriting recognition and using the television in the room, white boards or computer touch screens, placed on the wall facing the patient’s bed, to project relevant data from the EHR and share it with the patient and care team. Better mobile tools might also be useful, as would tools which meet teams’ needs during rounding and tracking of tasks.

The challenges identified in this study provide a basis for solutions on both system design and training levels. We suggest that solutions focus on three areas:

1. EHR design changes and interface improvement: It would be beneficial to consider different ways of visualizing data to prevent information overload and make the system easy to use in real time in the patient’s room. There is need to better integrate mobile devices that are easy to carry around between different floors/units in the hospital. It is equally important to consider integrating complementary HIT tools that support clinicians’ needs and workflow, such as handwriting recognition capabilities on mobile devices.
2. Hospital room adjustments and re-design: The current study site hospital room setting does not provide the infrastructure for sharing information between care team members or with the patient. Possible approaches include whiteboards that will allow projecting EHR data in the room, and positioning the bedside computer, so that it does not require the clinician to turn her/his back toward other clinicians in the room and the patient.
3. Care team training programs that consider EHR use during rounds: such programs need to address how the EHR can be better integrated into the workflow in ways that do not impede team communication, especially during rounds and promote EHR use for improving communication and information sharing between care team clinicians.

## Limitations

This study has several limitations. First, it involves a single site. Different workflows and use patterns may be observed in other settings. Second, as is typical in qualitative research, the sample size was not large. We attempted to address this limitation by conducting a mixed-methods approach including observations and semi-structured interviews as well as quantitative analysis of a user preference survey.

We recognize that some of the challenges described in this paper may arise from the EHR system being relatively new (implemented in May 2015). However, all clinicians who participated in the observations and interviews have had several years of experience with a different EHR system and at least 2.5 years of experience with the current one, if not more. We concluded that a 2.5 year use period would be a sufficient time to minimize learning curve issues.

## Conclusion

We evaluated an inpatient EHR, and found lack of standardized workflows, workarounds, team communication challenges, and system usability issues. Although EHRs can improve healthcare quality and have done so in many ways, our findings show that there are many challenges in the current inpatient environment that need to be addressed if EHRs are to reach their full potential.

## Supporting information

S1 Appendix Study fact sheet. (DOCX)

S2 Appendix Clinician semi-structured interview guide. (DOCX)

S3 Appendix Interview coding scheme. (DOCX)

S4 Appendix EMR integration into the inpatient workflow–user preference survey. (DOCX)
