# Supplementary material for: Assessing EHR use during hospital morning rounds: A multi-faceted study
Source: PLoS One. 2019 Feb 25;14(2):e0212816. doi: 10.1371/journal.pone.0212816 (PMC6388927; doi:10.1371/journal.pone.0212816)
Supplement: S1 Appendix — (DOCX) [file pone.0212816.s001.docx]

## S1 Appendix: Study fact sheet

Dear Sir/Madam,

My name is Dr. ** from ** Hospital and ** Medical School. We are studying clinicians' computer use during patient care in the Medical Wards.

We are conducting this study in order to find out how computer use affects care teams' communication with patients. We are also interested in better understanding care teams' and patients' information needs during rounds.

As part of our research, we are observing the care team's work and their interaction with patients during rounds as well as conducting interviews with care team members and patients. During the observations we plan to find out when, where, how and for what tasks computers are being used and how that use may affect the communication between patients and their care teams.

You may be asked to take part in the interview portion of the study as well. During the interviews, you will be asked several questions regarding your information needs during rounds and your view on how computer use influences the communication between patients and their care teams. If you agree, we would like to tape the interview for data analysis purposes.

We hope to use these findings to help improve healthcare information-system design and as result, better support care teams in their work and patients during their stay.

Please note that participation in this study is completely voluntary. All responses will be kept anonymous and confidential. Neither your name nor the name of your organization will be linked to your responses when we present our findings. Please feel free to skip any questions you think are not relevant to your experience.

You can call us with any questions or concern.

If you would like to speak with someone not directly involved in this study, please contact the Partners Human Research Committee office at: **

We thank you for your participation!
