# Supplementary material for: Assessing EHR use during hospital morning rounds: A multi-faceted study
Source: PLoS One. 2019 Feb 25;14(2):e0212816. doi: 10.1371/journal.pone.0212816 (PMC6388927; doi:10.1371/journal.pone.0212816)
Supplement: S2 Appendix — (DOCX) [file pone.0212816.s002.docx]

## S2 Appendix: Clinician semi-structured interview guide

- Please walk me through your workflow (from the beginning of your morning shift until after rounds are completed and you return to your office)
  - Main tasks before, during and after rounds
  - Do you have any interaction with the patients after morning rounds are completed? If so, please describe
- What information regarding the patient do you need available
  - Before entering the patient’s room
  - While in the patient’s room
  - After leaving the patient’s room
- Do you use the EHR
  - Before rounds
    - If yes, for what purposes and tasks?
  - During rounds
    - If yes, for what purposes and tasks?
  - After rounds
    - If yes, for what purposes and tasks?
- While in the patient's room do you access the EHR?
  - If yes, please elaborate on information input and output.

If the EHR is not accessed in the patient's room, please explain why
