# Supplementary material for: Assessing EHR use during hospital morning rounds: A multi-faceted study
Source: PLoS One. 2019 Feb 25;14(2):e0212816. doi: 10.1371/journal.pone.0212816 (PMC6388927; doi:10.1371/journal.pone.0212816)
Supplement: S3 Appendix — (DOCX) [file pone.0212816.s003.docx]

## S3 Appendix: Interview coding scheme

| **Interview Coding Scheme:** |
| --- |
| Device type |
| Use of paper: printouts, writing |
| Information needs (EHR-based) in pt's room |
| Workflow description |
| Early morning habits |
| Problems with current workflow |
| EHR use during rounds |
| Pre-rounding activities |
| Pre-rounding data needs |
| Post-rounding activities |
| Rounding order (prioritization) |
| EHR system evaluative comments |
| EHR system positive statement |
| EHR system critiques and problems |
| Discharge and IT/information resources for pts |
| Communication: EHR influence |
| Intra-rounding team |
| Patient-clinician |
| Patient-family engagement |
| Suggestions for future |
