# Supplementary material for: Assessing EHR use during hospital morning rounds: A multi-faceted study
Source: PLoS One. 2019 Feb 25;14(2):e0212816. doi: 10.1371/journal.pone.0212816 (PMC6388927; doi:10.1371/journal.pone.0212816)
Supplement: S4 Appendix — (DOCX) [file pone.0212816.s004.docx]

## S4 Appendix: EMR integration into the inpatient workflow – user preference survey

**EMR INTEGRATION into the inpatient workflow**

Dear participant,

The following 10-minute survey is part of an observational study that explores the integration of Epic's EMR into the care teams' workflow at Brigham and Women's Hospital.

The purpose of this survey is to establish a better understanding of your preferences when using the EMR.

We hope that this study will help generate the tools to better integrate healthcare information systems and patient-centered care in inpatient settings. Your input is extremely valuable in helping us achieve this goal.

Participation in the survey is voluntary and you are free to decide whether to participate. You may also opt out at any time.

All responses will remain anonymous.

Thank you for your participation!

Dr. Shiri Hassid
Email: [assishassid@g.harvard.edu](mailto:assishassid@g.harvard.edu)

Phone: 617-902-8261

1. What is your age

- 25-34 years old
- 35-44 years old
- 45-54 years old
- 55-64 years old
- 65 or older

1. What is your current medical position?

- Attending
- Resident
- Intern
- Medical Student
- Physician Assistant

1. Years of experience working with Epic: ____
2. When do you use the EMR while rounding on a patient? (please indicate all that apply)

- Before entering the patient's room
- In the patient's room
- After leaving the patient's room

1. Please rate the following statements regarding patient rounds, by checking (v) the response/s that best describe your workflow:

1. **When I review the patient's medical record on Epic I use a:**

|  | **Rating** | | | |
| --- | --- | --- | --- | --- |
| **Question** | **Never** | **Sometimes** | **Most of the time** | **Always** |
| Tablet |  |  |  |  |
| Smartphone |  |  |  |  |
| Computer on wheels (COW) |  |  |  |  |
| Computer at the nurses' station |  |  |  |  |
| Computer at the patient's bedside |  |  |  |  |

1. **When using Epic during rounds, do you share data from the patient's medical record?**

|  | **Rating** | | | |
| --- | --- | --- | --- | --- |
| **Question** | **Never** | **Sometimes** | **Most of the time** | **Always** |
| **Verbally** with the care team |  |  |  |  |
| **Verbally** with the patient/their family member |  |  |  |  |
| **Visually** with the care team (for example by turning the screen) |  |  |  |  |
| **Visually** with the patient/their family member (for example by turning the screen) |  |  |  |  |

1. **When a care team member uses Epic during rounds:**

|  | **Rating** | | | |
| --- | --- | --- | --- | --- |
| **Question** | **Never** | **Sometimes** | **Most of the time** | **Always** |
| I find that it interrupts communication **within the care team** |  |  |  |  |
| I find that it interrupts communication **with the patient/family member** |  |  |  |  |

1. **I find that using Epic during rounds is helpful/useful as a tool for:**

|  | **Rating** | | | |
| --- | --- | --- | --- | --- |
| **Question** | **Never** | **Sometimes** | **Most of the time** | **Always** |
| Explaining the patient's medical condition to the patient and/or his family member |  |  |  |  |
| Synchronizing the care team regarding the patients |  |  |  |  |
| Being efficient during rounds |  |  |  |  |
| Teaching purposes |  |  |  |  |

Comments:

_____________________________________________________________________
